# Detection of *Burkholderia pseudomallei* with CRISPR-Cas12a based on specific sequence tags

**DOI:** 10.3389/fpubh.2023.1153352

**Published:** 2023-04-28

**Authors:** Jia-Xin Zhang, Jian-Hao Xu, Bing Yuan, Xiao-Dong Wang, Xu-hu Mao, Jing-Lin Wang, Xiang-Li-Lan Zhang, Yuan Yuan

**Affiliations:** ^1^State Key Laboratory of Pathogen and Biosecurity, Beijing Institute of Microbiology and Epidemiology, Beijing, China; ^2^School of Life Sciences, Fujian Agriculture and Forestry University, Fuzhou, Fujian, China; ^3^Department of Clinical Microbiology and Immunology, The Third Military Medical University, Chongqing, China

**Keywords:** *Burkholderia pseudomallei*, specific sequence tags, CRISPR-Cas12a, visual detection, species discrimination

## Abstract

Melioidosis is a bacterial infection caused by *Burkholderia pseudomallei* (*B. pseudomallei*), posing a significant threat to public health. Rapid and accurate detection of *B. pseudomallei* is crucial for preventing and controlling melioidosis. However, identifying *B. pseudomallei* is challenging due to its high similarity to other species in the same genus. To address this issue, this study proposed a dual-target method that can specifically identify *B. pseudomallei* in less than 40 min. We analyzed 1722 *B. pseudomallei* genomes to construct large-scale pan-genomes and selected specific sequence tags in their core genomes that effectively distinguish *B. pseudomallei* from its closely related species. Specifically, we selected two specific tags, LC1 and LC2, which we combined with the Clustered Regularly Interspaced Short Palindromic Repeats (CRISPR)-CRISPR associated proteins (Cas12a) system and recombinase polymerase amplification (RPA) pre-amplification. Our analysis showed that the dual-target RPA-CRISPR/Cas12a assay has a sensitivity of approximately 0.2 copies/reaction and 10 fg genomic DNA for LC1, and 2 copies/reaction and 20 fg genomic DNA for LC2. Additionally, our method can accurately and rapidly detect *B. pseudomallei* in human blood and moist soil samples using the specific sequence tags mentioned above. In conclusion, the dual-target RPA-CRISPR/Cas12a method is a valuable tool for the rapid and accurate identification of *B. pseudomallei* in clinical and environmental samples, aiding in the prevention and control of melioidosis.

## 1. Introduction

Melioidosis is a tropical disease caused by the aerobic, Gram-negative motile bacillus which is classified as a category B biological agent by the Centers for Disease Control and Prevention (CDC) of America (1, 2). It is a highly pathogenic endemic zoonotic disease in many tropical countries, particularly in Southeast Asia and Northern Australia. In China, the southern regions of Hainan, Guangdong, Guangxi, and Fujian are the endemic areas for the disease. Hainan carries the most significant burden compared to other provinces, and residing in or traveling to this tropical island is an important risk factor for infection (3). Hainan also experienced a geographical melioidosis outbreak in Hainan following the 2021 typhoon (4). Epidemiological studies have indicated that melioidosis often affects individuals with one or more pre-existing conditions associated with an altered immune response, such as diabetes, compromised liver or decreased renal function appears to have an increased risk of infection (5). The most severe clinical symptom is sepsis, a life-threatening, dysregulated, systemic inflammatory and immune response that can cause organ dysfunction with a case fatality rate of up to 40%. The number of cases worldwide is estimated to be 165,000 per year, of which 89,000 are fatal (6).

The *B. pseudomallei* strain K96243 has two chromosomes with significant functional partitioning of genes. The large chromosome has 4.07 Mbp and carries many core functions related to central metabolism and cell growth. The small chromosome, with 3.17 Mbp, encodes accessory functions associated with adaptation and survival in different niches. The genome has 7,232 protein coding sequences (CDS), 60 transfer RNA (tRNA) genes, and 12 ribosomal RNA (rRNA) genes. Approximately 6% of the genome consists of putative genomic islands that are likely obtained by horizontal gene transfer (7). There are over 40 species of *Burkholderia* in the genus, among which *B. pseudomallei* and *Burkholderia mallei* (*B. mallei*), are the most pathogenic. However, they are very similar in genetic and immunological features. Previously, randomly amplified polymorphic DNA (RAPD) (8),16S rRNA gene sequencing (9), multilocus sequence typing (MLST) (10), polymerase chain reaction-restriction fragment length polymorphism (PCR–RFLP) (11), probe-based real-time PCR and loop-mediated isothermal amplification targeting *Burkholderia* type III secretion system genes (12, 13), multiplex PCR assays (14–16), single-nucleotide polymorphism (SNP) typing (17), DNA microarrays (18) and proteome profiling (19) have been developed to detect and differentiate *B. pseudomallei*. Unfortunately, the specificity and coverage of the above primers and probes for *B. pseudomallei* were found to be insufficient after NCBI BLAST website verification. To address these shortcomings, this study aimed to identify unique tags based on genome differences between different species and genera of pathogens, as well as their intraspecific polymorphisms.

Recombinase polymerase amplification (RPA) is a thermostatic amplification technology that can expand target DNA to detectable levels in 10 min in an isothermal reaction condition (20). Clustered regularly interspaced short palindromic repeats (CRISPR)—CRISPR associated proteins 12a (Cas12a), a powerful diagnostic tool, has been widely used for the detection of pathogenic bacteria in recent years (21, 22). When the CRISPR-Cas12a system is used to establish a sensing platform for the detection of pathogenic bacteria, Cas12a-crRNA can recognize target DNA and activate the trans-cleavage of Cas12a which will cleave the non-target single-stranded DNA (electrochemistry, fluorescence probe, etc.) (23, 24). Therefore, we can use this feature to develop detection methods *in vitro*.

**Figure 1:**
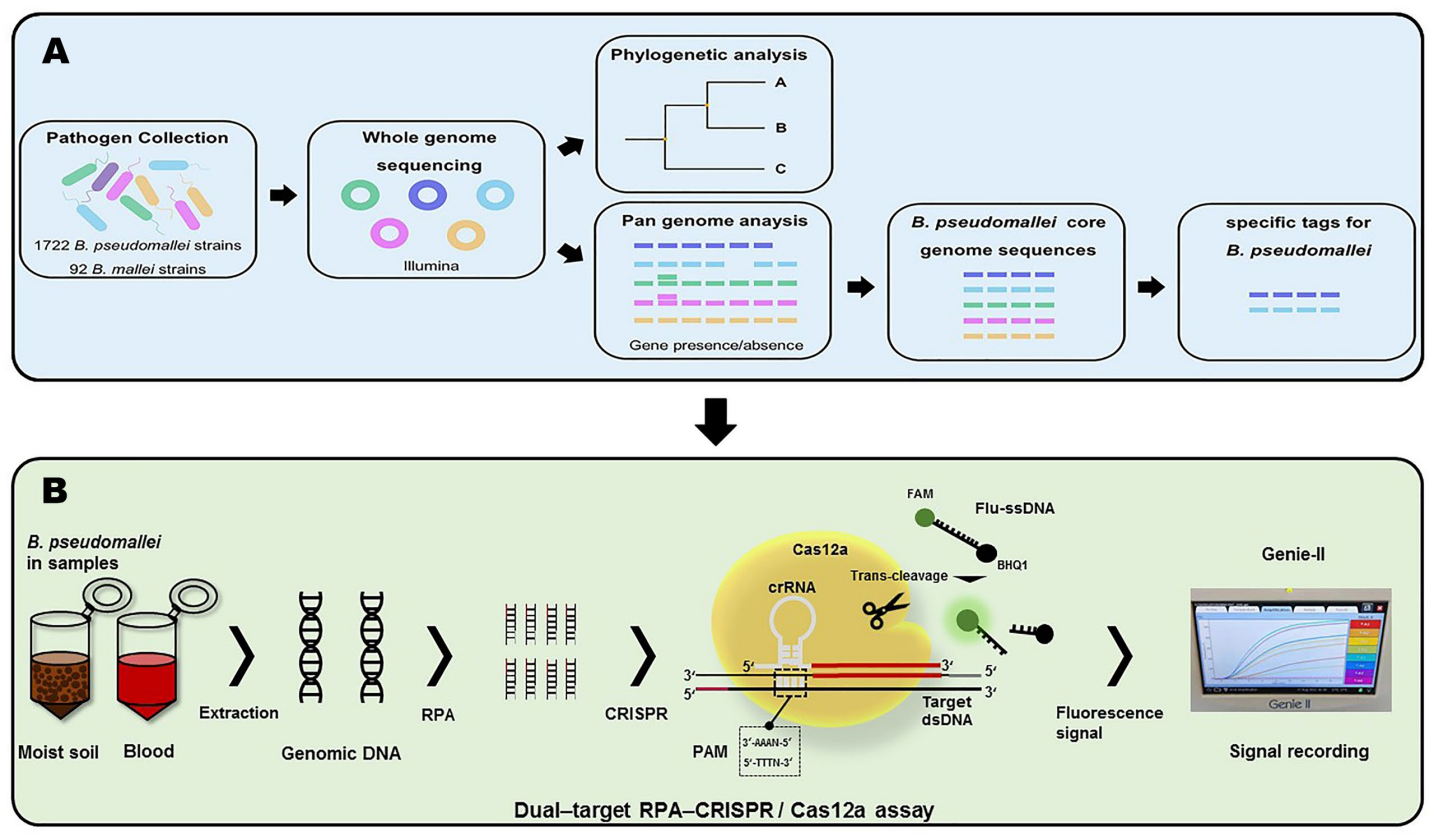
Workflow of this project. **(A)** Acquisition of *B. pseudomallei*-specific tags. **(B)** Schema of assay process for the detection of *B. pseudomallei* with the dual–target RPA–CRISPR/Cas12a assay.

In this study, we constructed the pan-genome and evaluated gene presence/absence from the genomic sequences of 1722 *B. pseudomallei* strains and 92 *B. mallei* strains using Roary software. Subsequently, 44 specific sequence tags for quick identification of *B. pseudomallei* were found from the core genome sequences (Figure 1A). The specific tags, containing protospacer adjacent motif (PAM) sequences, of *B. pseudomallei* were selected. Finally, two of the newly developed *B. pseudomallei*-specific tags, Cas12a as a biosensor coupled with RPA pre-amplification and fluorescent signal output, were used to construct the dual-target RPA-CRISPR/Cas12a assay for rapid, sensitive, and specific detection of *B. pseudomallei* (Figure 1B).

## 2. Materials and methods

### 2.1. Bacterial strains

A total of 1,722 *B. pseudomallei* genomes (13 newly sequenced and 1709 public) and 92 *B. mallei* genomes (9 newly sequenced and 83 public) were used in this work. All newly sequenced strains were derived from Beijing Institute of Microbiology and Epidemiology. The genomes of the newly sequenced strains had been uploaded to NCBI (The Bioproject number is PRJNA930628).^1^ The publicly available genomes were downloaded from NCBI (*B. pseudomallei*^2^; *B. mallei*).^3^

The genomic DNA of *Brucella melitensis*, *Brucella abortus*, *Brucella suis*, *Brucella canis*, *Francisella tularensis*, *Bacillus anthracis*, *Yersinia pestis*, *Burkholderia cepacian*, *Vibrio cholerae*, *Staphylococcus aureus*, *Vibrio vulnificus*, *Vibrio parahaemolyticus*, and *Salmonella typhi* provided by Beijing Institute of Microbiology and Epidemiology were used for the specificity tests.

### 2.2. Treatment of strains

*Burkholderia pseudomallei*, *B. mallei*, *B. melitensis*, *B. abortus*, *B. suis*, *B. canis*, *F. tularensis*, *B. anthracis*, and *Y. pestis* were cultured in biosafety level 3 (BSL-3) and subsequently heat inactivated. For safety, the inactivated bacteria were recoated, and no bacterial growth was found. Then, the inactivated bacteria were taken out of BSL-3. *Vibrio cholerae*, *Staphylococcus aureus*, *Vibrio vulnificus*, *Vibrio parahaemolyticus*, and *Salmonella typhi* were cultured in biosafety level 2 (BSL-2). Subsequently, the QIAamp^TM^ DNA Mini Kit (Qiagen, Germany) was used to extract genomic DNA from all the bacteria. Extracted DNA was stored at 4°C for a short time or at −40°C for longer periods.

### 2.3. Sequencing and assembly

Whole genome *de novo* sequencing was performed using Illumina MiSeq (Illumina, San Diego, CA, United States) to generate multiplexed paired-end libraries with an average insert size of 300 bp. Next, the raw short-read sequences of each strain were filtered for low-quality data using the FASTQ Quality Filter module in the FASTX-Toolkit software^4^ (25). Then, the filtered reads were assembled using the SPAdes 3.0-based software Shovill version 1.0.4^5^ (26) with default settings. The average genome size and GC content of all sequenced strains were 7.11 Mbp and 68.22%, respectively. The detailed description of assembly results was provided in Supplementary Table S1.

### 2.4. Phylogenetic analysis

The SNPs were identified through pairwise comparisons of 11 previously published *Burkholderia* genomes (*Burkholderia cenocepacia* J2315, *Burkholderia cenocepacia* HI2424, *Burkholderia ambifaria* AMMD, *Burkholderia dolosa* AUO158, *Burkholderia ubonensis* MSMB22, *Burkholderia ubonensis* Bu, *Burkholderia pseudomallei* K96243-1, *Burkholderia pseudomallei* K96243-2, *Burkholderia mallei* ATCC 23344, *Burkholderia mallei* SAVP1, and *Burkholderia thailandensis* E264) using MUMmer 3.0^6^ (27). Then, SNPs in repeated regions with low-quality scores (<20) or supported by few reads (<10 paired-end reads) were eliminated. The maximum likelihood tree (MLTree) was built using RaxML^7^ (28) based on the concatenated SNPs.

### 2.5. Screening for *Burkholderia pseudomallei*-core genome sequences

We annotated 1722 genomes of *B. pseudomallei* and 92 genomes of *B. mallei* using Prokka^8^ (29). The GFF3 files of *B. pseudomallei* and *B. mallei* generated by Prokka were then used in Roary^9^ (30) (parameter settings: -cd 100 -i 90 -e -mafft -p 4 -r -t 11) to identify the pan-genome and gene presence/absence. The unique core genome of *B. pseudomallei*, core base pairs/genes found in all strains of *B. pseudomallei* but not present in the pan-genome of *B. mallei*, were screened for subsequent analysis using an in-house Perl script (Supplementary Table S2).

### 2.6. Constructing specific sequence tags for *Burkholderia pseudomallei* based on the core genome sequences

To verify the specificity of the sequence tags, the core genome sequences of *B. pseudomallei* were further aligned using a local version of the NCBI BLASTN software and NCBI BLAST website.^10^ The identified sequence tags were only aligned to the genome sequences of *B. pseudomallei*, with 100% coverage and identity with all 1722 *B. pseudomallei* genome sequences. We then selected one specific sequence tag, containing PAM sequences for Cas 12a, on each of the two chromosomes of *B. pseudomallei*. These two specific sequence tags were named LC1 and LC2, and corresponding PCR primers were designed and synthesized.

### 2.7. The design and screening of RPA primers, RPA probes, and crRNA

The pre-amplification efficiency of RPA is an important for the detection sensitivity. RPA primers (LC1-F1 ~ F4/LC1-R1 ~ R4 and LC2-F1 ~ F4/LC2-R1 ~ R4) were designed by Primer Premier 6.0 according to the assay design manual of the TwistAmp^™^ DNA amplification kits. RPA probes LC1-P and LC2-P were designed from the amplified sequences of LC1-F/R and LC2-F/R, respectively. These candidate primers were screened with the same concentrations of positive reference plasmid (pEASY-T1-LC1 and pEASY-T1-LC2) as template DNA using the real-time RPA method, performed according to the TwistAmp^™^ exo Kit (Cambridge, United Kingdom) Quick Guide at 39°C for 10 min in a Genie-II (OptiGene, United Kingdom).

The crRNA spacer sequences were designed downstream of the PAM sequence containing 5′-TTTN-3′ on the RPA amplified sequence. The direct repeat sequence was added upstream of the crRNA spacer sequences (31). Particularly, LC1-crRNA-1 and LC1-crRNA-2 were designed by the amplified sequence of LC1-F/R, whereas LC2-crRNA-1 and LC2-crRNA-2 were designed by the amplified sequence of LC2-F/R. Subsequently, the fluorescent single-stranded DNA reporter (Flu-ssDNA) modified with fluorophore 6-FAM and quencher BHQ1 was trans-cleaved by Cas12a, which allowed the identification of the presence or absence of the target genes LC1 and LC2. The RPA primers, RPA probes, crRNA, and Flu-ssDNA were synthesized by Shanghai Sangon Biotech Co., Ltd. (China). Details of the oligonucleotides were listed in Table 1.

**Table 1:** The sequences of RPA primers, RPA probes, and crRNA.

Name	Sequence (5′–3′)
LC1-F1	GGGTTTCCAGAAGGGCTGCAAGCACCAAATG
LC1-F2	CGGGTTTCCAGAAGGGCTGCAAGCACCAAAT
LC1-F3	GGTTTCCAGAAGGGCTGCAAGCACCAAATGT
LC1-F4	GTTTCCAGAAGGGCTGCAAGCACCAAATGTG
LC1-R1	GCAATGTCTTACAACAAGCCATGCCGTCATCTTCA
LC1-R2	CAATGTCTTACAACAAGCCATGCCGTCATCTTCAT
LC1-R3	TGCAATGTCTTACAACAAGCCATGCCGTCATCTTC
LC1-R4	ATGCAATGTCTTACAACAAGCCATGCCGTCATCTT
LC2-F1	GAGGCTCGAACAACGTCGGCTTCCCAGGAT
LC2-F2	AGAGGCTCGAACAACGTCGGCTTCCCAGGA
LC2-F3	GAGAGGCTCGAACAACGTCGGCTTCCCAGG
LC2-F4	AGGCTCGAACAACGTCGGCTTCCCAGGATT
LC2-R1	AATGAATTCGTCGGCACGCGCCAGCCAAAT
LC2-R2	ATGAATTCGTCGGCACGCGCCAGCCAAATG
LC2-R3	CAATGAATTCGTCGGCACGCGCCAGCCAAA
LC2-R4	ACAATGAATTCGTCGGCACGCGCCAGCCAA
LC1-P	TCGCATCCGCCGACCGATTTGATGTTAATGTCGTTACGAAAGACGAGCA
LC2-P	TTCATATCGAACTTAACTGATTCAGAGAAATACTCAATCTCGGAAAATA
LC1-crRNA-1	UAAUUUCUACUAAGUGUAGAUGCAUCCGCCGACCGAUUUGATGUU
LC1-crRNA-2	UAAUUUCUACUAAGUGUAGAUAUGUUAAUGUCGUUACGAAAGACG
LC2-crRNA-1	UAAUUUCUACUAAGUGUAGAUCGCUACACCAUAGCAGUGUUCGCG
LC2-crRNA-2	UAAUUUCUACUAAGUGUAGAUAUAUCGAACUUAACUGAUUCAGAG
Flu-ssDNA	6-FAM-CCCCCCCCCCCC-BHQ1

The unified sequence (UAAUUUCUACUAAGUGUAGAU) is the direct repeat sequence (known as a 5′ handle), and the long underlined sequence is the spacer sequence (guide sequence).

### 2.8. RPA-CRISPR/Cas12a assay

Template DNAs were amplified for 30 min in a Genie-II at 39°C according to the TwistAmp^™^ Basic Kit (Cambridge, United Kingdom) Quick Guide to obtain RPA amplification products. The following CRISPR reaction system was composed of 40 μL reaction solution: 0.3 μL Cas12a (75 nM), 2 μL Flu-ssDNA (500 nM), 0.5 μL RNase inhibitor, 3 μL NEBuffer3.1, 10 μL crRNA (500 nM) and 4.2 μL double-distilled water (ddH_2_O). LbCas12a protein, NEBuffer 3.1, and RNA inhibitor were provided by NEW ENGLAND BioLabs, Inc. (United States). DNase/RNase-free distilled, deionized water (ddH_2_O) was provided by Tiangen Biochemical Co., Ltd. Positive reference plasmids for *B. pseudomallei* detection (pEASY-T1-LC1 and pEASY-T1-LC2) were constructed by our lab. Finally, we took 20 μL of the RPA-amplified product as a template, and then ran the CRISPR/Cas12a reaction system for 10 min at 37°C in the Genie-II.

### 2.9. Evaluation of the sensitivity and specificity

After identifying the best RPA primers and crRNAs, the sensitivity of the dual-target RPA-CRISPR/Cas12a assay was evaluated. Sensitivity was tested by gradually diluting 2 μL (0.1 ~ 100 copies/μL) of two positive plasmids (0.2 ~ 200 copies/reaction), and 2 μL (1 ~ 100 fg/μL) of *B. pseudomallei* genomic DNA (2 ~ 200 fg) were used as template DNA to test the sensitivity.

The specificity of the dual-target RPA-CRISPR/Cas12a assay for *B. pseudomallei* was investigated with low input *B. pseudomallei* genomic DNA (100 fg) as well as high input *B. mallei* genomic DNA (100 pg) and 12 non-*B. pseudomallei* bacterial genomic DNA (100 pg). DDH_2_O was used as a no-template control (NTC). Twelve non-*B. pseudomallei* bacterial genomic DNA were prepared by mixing the genomic DNA of 12 other pathogenic bacteria, including *B. melitensis*, *B. abortus*, *B. suis*, *B. canis*, *F. tularensis*, *B. anthracis*, *Y. pestis*, *V. cholerae*, *S. aureus*, *V. vulnificus*, *V. parahaemolyticus*, and *S. typhi*.

### 2.10. Simulated blood and moist soil samples test by RPA-CRISPR/Cas12a assay

As *B. pseudomallei* bacteria are highly pathogenic and must be handled under BSL-3 conditions (Manual of Clinical Microbiology, 8th ed., American Society for Microbiology, Washington, DC), we only use the genomic DNA to prepare test samples.

To verify the feasibility of the dual-target RPA-CRISPR/Cas12a assay for *B. pseudomallei*, a total of 12 blood samples and 12 moist soil samples were collected. In a double-blinded test, different concentrations of *B. pseudomallei* genomic DNA (between 200 and 10 fg/μL), *B. mallei* genomic DNA (1 × 10^5^ fg/μL), and *B. cepaci*a genomic DNA (1 × 10^5^ fg/μL), as well as a blank control (BC) sample with ddH_2_O, were added. DNA was extracted with the QIAamp DNA Mini kit, and 2 μL nucleic acid extract was used for real-time PCR (RT-PCR) and the dual-target RPA-CRISPR/Cas12a assay for *B. pseudomallei*. The RT-PCR assay for *B. pseudomallei* was performed according to a previously published method (32) in the qTOWER3G instrument (Analytikjena, Germany) with the following program: pre-denaturation at 95°C for 2 min, followed by 45 cycles of denaturation at 95°C for 5 s and annealing and extension at 56°C for 10 s, 72°C for 10 s, and 40°C for 20 s. The results of the RT-PCR and the dual-target RPA-CRISPR/Cas12a assays for *B. pseudomallei* were then analyzed and compared.

### 2.11. Data and statistical analysis

The 10 min fluorescence signals of each group were collected from Genie-II. The experimental data of each group was normalized to create an intuitive heat map. At the same template DNA concentration, a profound color and a normalized value closer to 1.00 are indicative of a greater fluorescence signal. The fold change value (FCV) in the fluorescence of each group was calculated as the average fluorescence value of each testing group divided by the average fluorescence signal of the no-template control (NTC) group.

## 3. Results

### 3.1. Validation of published primers and probes for *Burkholderia pseudomallei*

According to the results of NCBI BLAST website verification, we found that the published primers and probes for *B. pseudomallei* were insufficiently specific and covered (Supplementary Table S3).

### 3.2. Acquisition of *Burkholderia pseudomallei*-core genome sequences

This study analyzed the whole genomes of 1722 of *B. pseudomallei* strains and 92 *B. mallei* strains isolated between 1996 and 2019, from six continents and more than 40 countries.

The construction of phylogenetic trees shows genetic distances and relationships between individuals within a population and is therefore useful for studying population structure and species evolution. Phylogenetic analysis demonstrated that *B. pseudomallei* and *B. mallei* were the most closely related but still clearly distinguishable (Supplementary Figure S1).

**Figure 2:**
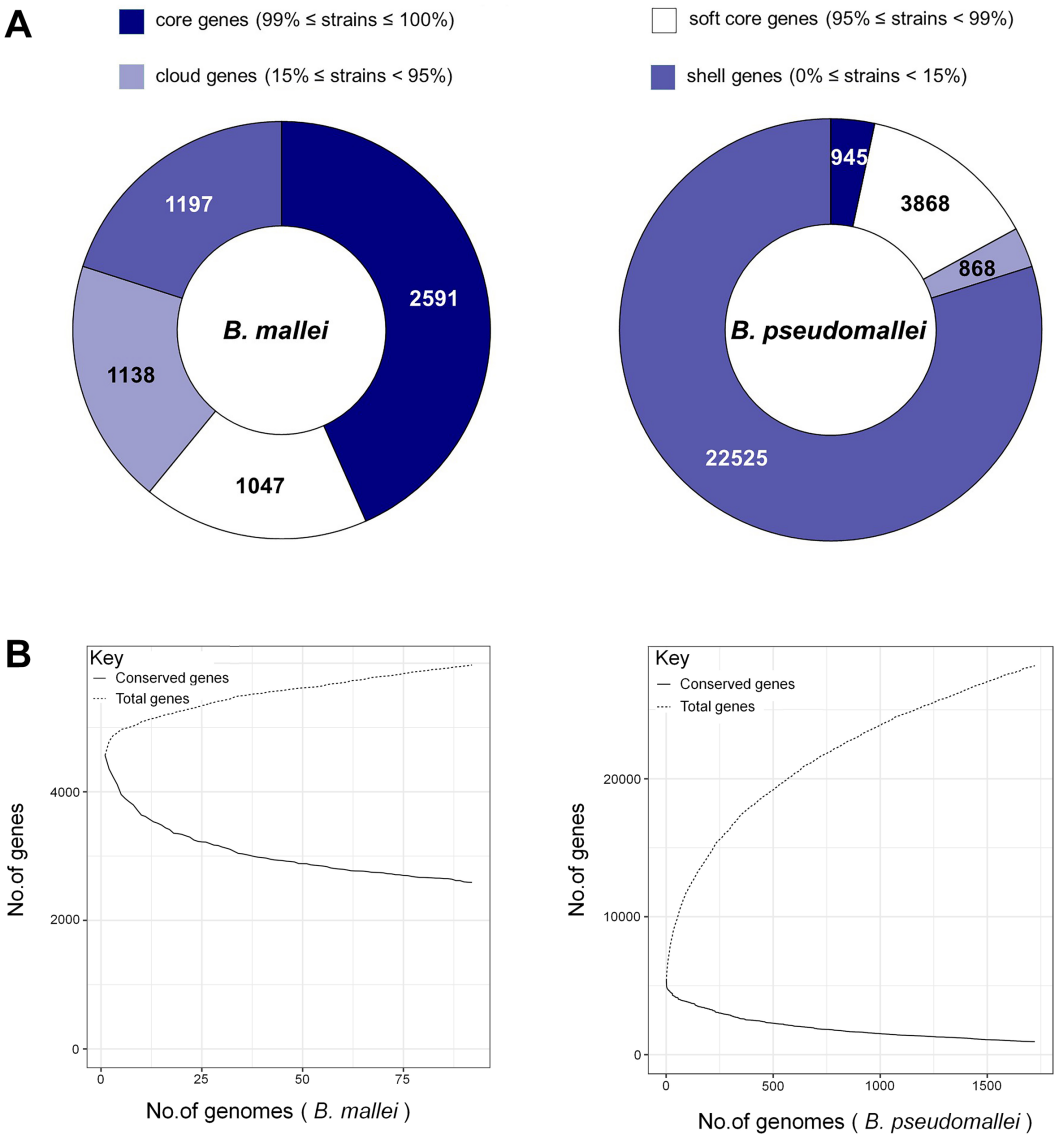
Pan-genome analysis of *B. pseudomallei* and *B. mallei.* **(A)** Number of gene families (*B. pseudomallei* and *B. mallei*). **(B)** The trend chart of the size of core-pan genes (*B. pseudomallei* and *B. mallei*).

The numbers of gene families for *B. pseudomallei* and *B. mallei* were calculated using MUMmer and Roary. From the total of 28,206 genes in the *B. pseudomallei* pan-genome, we identified 945 core genes (99% ≤ strains≤100%), 3,868 soft core genes (95% ≤ strains<99%), 868 shell genes (15% ≤ strains<95%) and 22,525 cloud genes (0% ≤ strains<15%). Of the 5,973 genes of the *B. mallei* pan-genome, we identified 2,591 core genes, 1,047 soft core genes, 1,197 shell genes, and 1,138 cloud genes (Figure 2A). As the number of strains increased, the number of core genes gradually decreased, and the number of pan genes increased, suggesting that the genetic material of *B. pseudomallei* and *B. mallei* is still “open” with high genetic diversity (Figure 2B).

We also identified unique core genome sequences of *B. pseudomallei* using an in-house Perl script (Figure 1A).

### 3.3. Obtaining *Burkholderia pseudomallei*-specific tags

**Figure 3:**
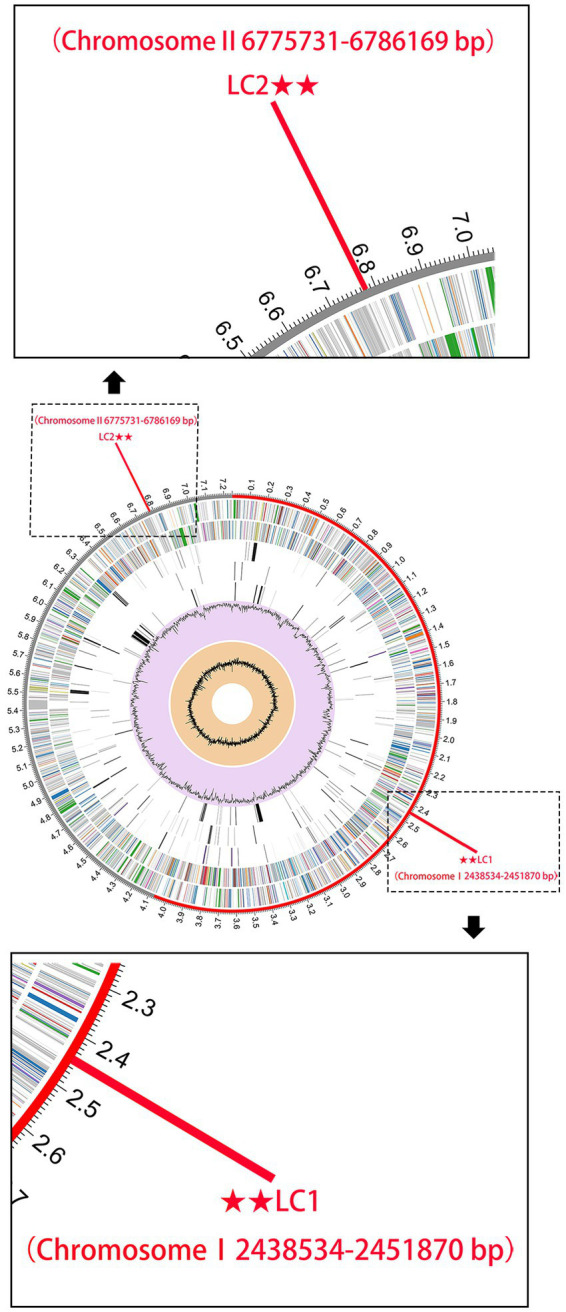
The genome annotations of *B. pseudomallei* K96243. This includes, from outer to inner rings, the distribution of the 2 *B. pseudomallei*-specific tags (for subsequent CRISPR-Cas12a analysis), the contigs, CDS on the forward strand, CDS on the reverse strand, RNA genes, CDS with homology to known antimicrobial resistance genes, CDS with homology to known virulence factors, GC content, and GC skew.

Sequences fragments on the *B. pseudomallei* core genome with a length of >5,000 bp were chosen to boost detection specificity. In total, 44 *B. pseudomallei*-specific sequence tags were screened using a local version of the NCBI BLASTn and online NCBI BLAST that contain all the public genomes up to date (Supplementary Table S4). The results showed that all the 44 *B. pseudomallei*-specific sequence tags were identical to the sequences of the *B. pseudomallei* species with query cover and sequence identity being both 100%. Also, The *B. pseudomallei*-specific sequence tags were inconsistent with non-*B. pseudomallei* species and strains and could therefore be used to identify *B. pseudomallei*. We selected two *B. pseudomallei*-specific sequence tags to detect *B. pseudomallei* and named them LC1 and LC2 sites (Figure 3).

### 3.4. Optimal RPA primers and crRNAs

**Figure 4:**
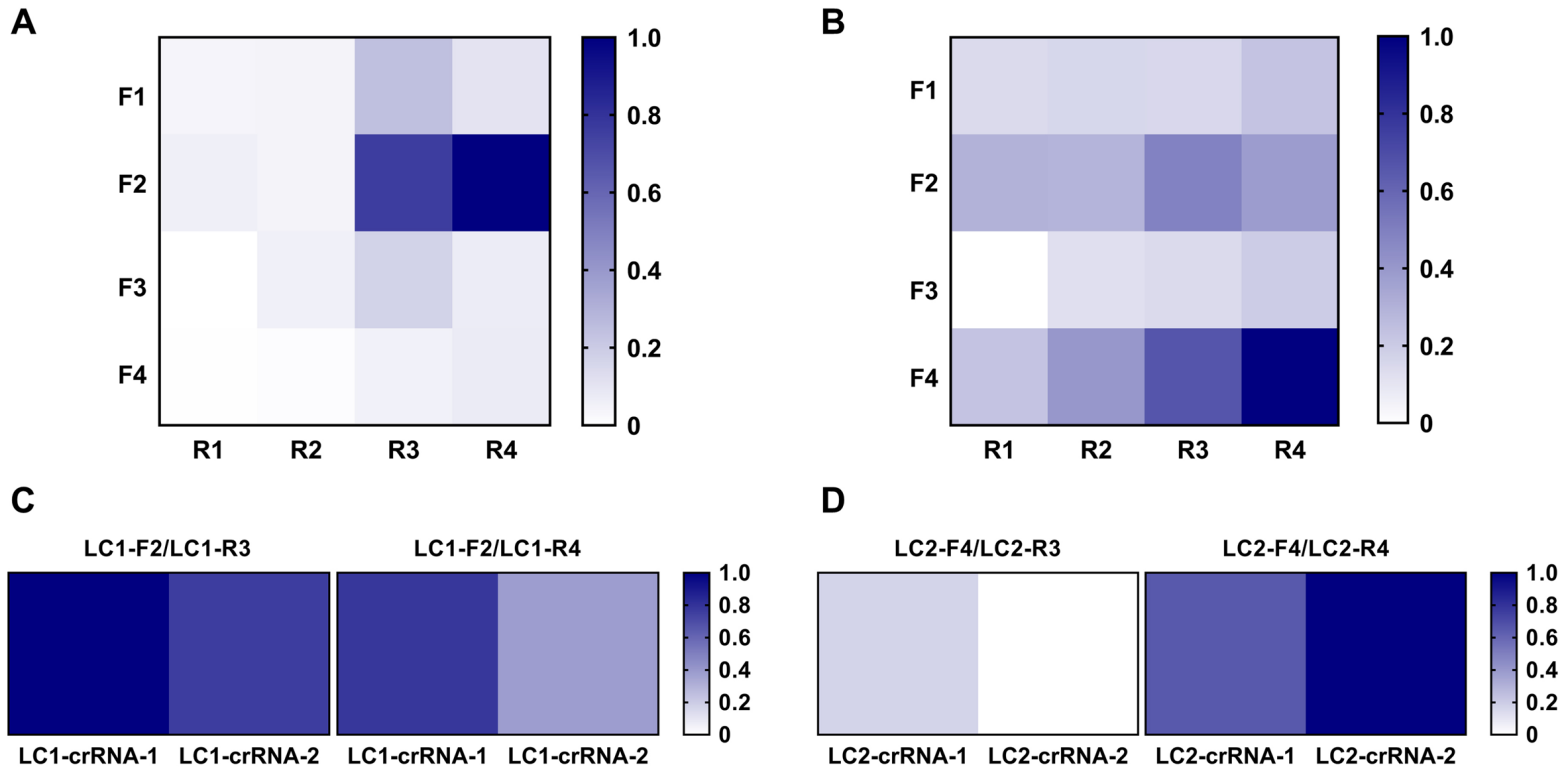
Screening of optimal RPA primers and crRNAs. **(A)** LC1 RPA primer screening using the same concentration of DNA template. **(B)** LC2 RPA primer screening using the same concentration of DNA template. **(C)** Identifying the best LC1 RPA primer and crRNA. **(D)** Identifying the best LC2 RPA primer and crRNA.

Initially, RPA fluorescent probes LC1-P and LC2-P were used to cross-screen the designed RPA primers LC1-F1 ~ F4/LC1-R1 ~ R4 and LC2-F1 ~ F4/LC2-R1 ~ R4. The screening results for the LC1-RPA primers were shown in Figure 4A. Primers LC1-F2/R3 (0.76) and LC1-F2/R4 (1.00) had preferably normalized values. The screening results for the LC2-RPA primers were shown in Figure 4B. Primers LC2-F4/R3 (0.66) and LC2-F4/R4 (1.00) had preferably normalized values.

After preliminary screening of RPA primers, crRNA was designed to construct the CRISPR/Cas12a system, and RPA was used for pre-amplification to establish the RPA-CRISPR/Cas12a method. The candidate primers LC1-F2/R3 and LC1-F2/R4 were combined with LC1-crRNA-1 and LC1-crRNA-2, respectively. The candidate primers LC2-F4/R3 and LC2-F4/R4 were combined with LC2-crRNA-1 and LC2-crRNA-2, respectively. Two positive reference plasmids (10 copies/μL, 2 μL) were used as template DNA and the primer, combinations were screened with the RPA-CRISPR reaction. For LC1, the primer LC1-F2/R3 combined with LC1-crRNA-1 had the greatest normalized value (Figure 4C). For LC2, the primer LC2-F4/R4 combined with LC2-crRNA-2 had the greatest normalized value and was therefore used to establish the LC2 RPA-CRISPR/Cas12a assay for *B. pseudomallei* (Figure 4D).

### 3.5. Evaluation of the sensitivity and specificity of the dual-target RPA-CRISPR/Cas12a assay for *Burkholderia pseudomallei*

**Figure 5:**
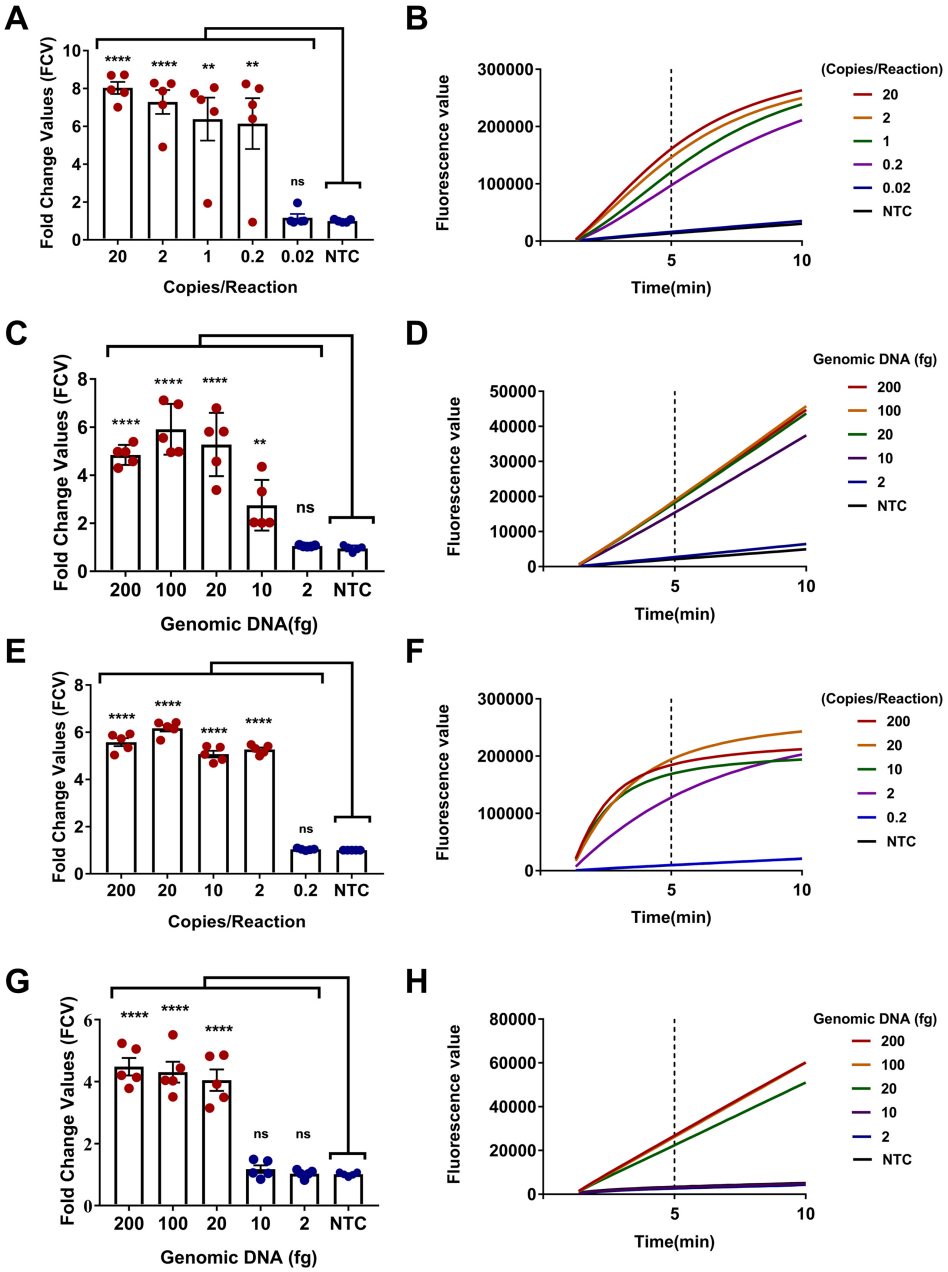
Sensitivity evaluation of the dual-target RPA-CRISPR/Cas12a assay. **(A,B)** Positive reference plasmids and **(C,D)** *B. pseudomallei* genomic DNA, LC1 RPA-CRISPR/Cas12a assay. **(E,F)** Positive reference plasmids and **(G,H)** *B. pseudomallei* genomic DNA, LC2 RPA-CRISPR/Cas12a assay. Error bars represent mean ± SEM, where *n* = 5 replicates, matched samples *t*-test, ****(*p* < 0.0001); **(A)** **(1 copies/reaction, *p* = 0.0015; 0.2 copies/reaction, *p* = 0.0050); **(C)** **(10 fg, *p* = 0.0054); ns (non-significant).

The sensitivity of the dual-target RPA-CRISPR/Cas12a assay was evaluated. Obviously, two assays completed the detection of target DNA within 40 min, requiring 30 min for RPA and 10 min for CRISPR (Figures 5B,D,F,H). The LC1 RPA-CRISPR/Cas12a assay showed a low detection limit for *Burkholderia pseudomallei* at 0.2 copies/reaction and 10 fg genomic DNA (Figures 5A–D). LC2 RPA-CRISPR/Cas12a assay showed a low detection limit for *B. pseudomallei* down to 2 copies/reaction and 20 fg genomic DNA (Figures 5E–H).

**Figure 6:**
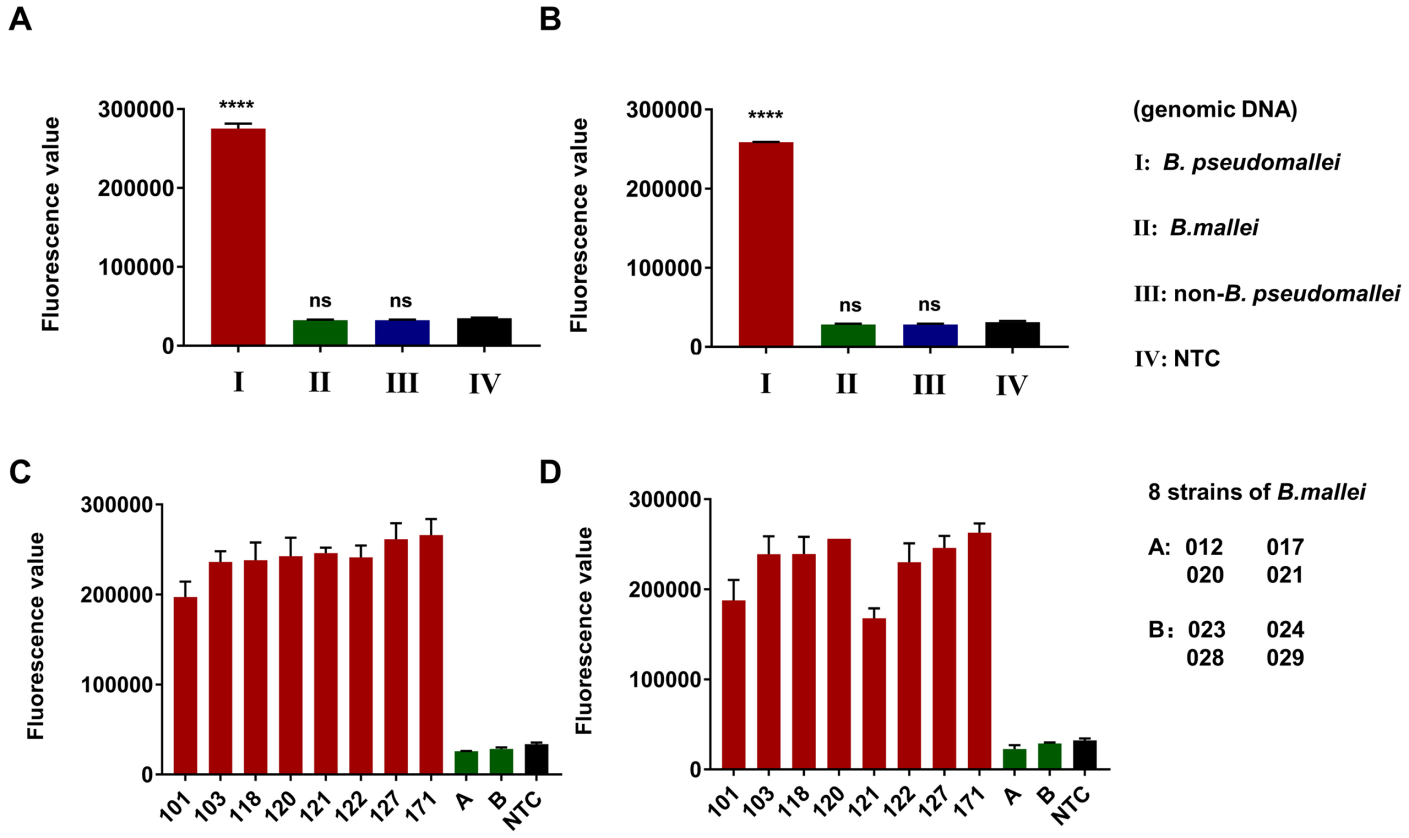
Specificity evaluation of dual-target RPA-CRISPR/Cas12a assay, Error bars represent mean ± SEM, where *n* = 4 replicates, matched samples *t*-test, ****(*p* < 0.0001), ns (non-significant), LC1 **(A,C)** and LC2 **(B,D)**.

The specificity of the dual-target RPA-CRISPR/Cas12a assay for *B. pseudomallei* was investigated with low input *B. pseudomallei* genomic DNA (100 fg) as well as high input *B. mallei* genomic DNA (100 pg) and 12 non-*B. pseudomallei* bacterial genomic DNA (100 pg). DDH_2_O was used as a no-template control (NTC). As shown in Figures 6A,B, the dual-target RPA-CRISPR/Cas12a assay was only positive for *B. pseudomallei*. In addition, for testing 8 strains of *B. pseudomallei* (101, 103, 118,120, 121, 122, 127, 171) and 8 strains of *B. mallei* (012, 017, 020, 021, 023, 024, 028, 029) (Figures 6C,D), the dual-target RPA-CRISPR/Cas12a assay distinguishes specific detection of *B. pseudomallei*.

### 3.6. Simulated blood and moist soil samples teste by RPA-CRISPR/Cas12a assay

After completion of the sensitivity and specificity evaluations, we analyzed the clinical adaptation feasibility of the dual-target assay using clinical samples, derived from human blood, and environmental samples, derived from naturally moist soil.

Concomitantly, RT-PCR was used as an auxiliary reference experiment. We analyzed the feasibility of the RT-PCR assay and used it to quantify the genomic DNA spiked into the simulated samples. A dilution gradient of *B. pseudomallei* genomic DNA (1 ng, 100, 10, 1 pg., and 100 fg) in ddH_2_O for constructing the standard curve of the RT-PCR assay, while the ddH_2_O was set to NTC synchronously (Supplementary Figure S2), R^2^ > 0.99, Y = 3.182*X + 23.56.

**Figure 7:**
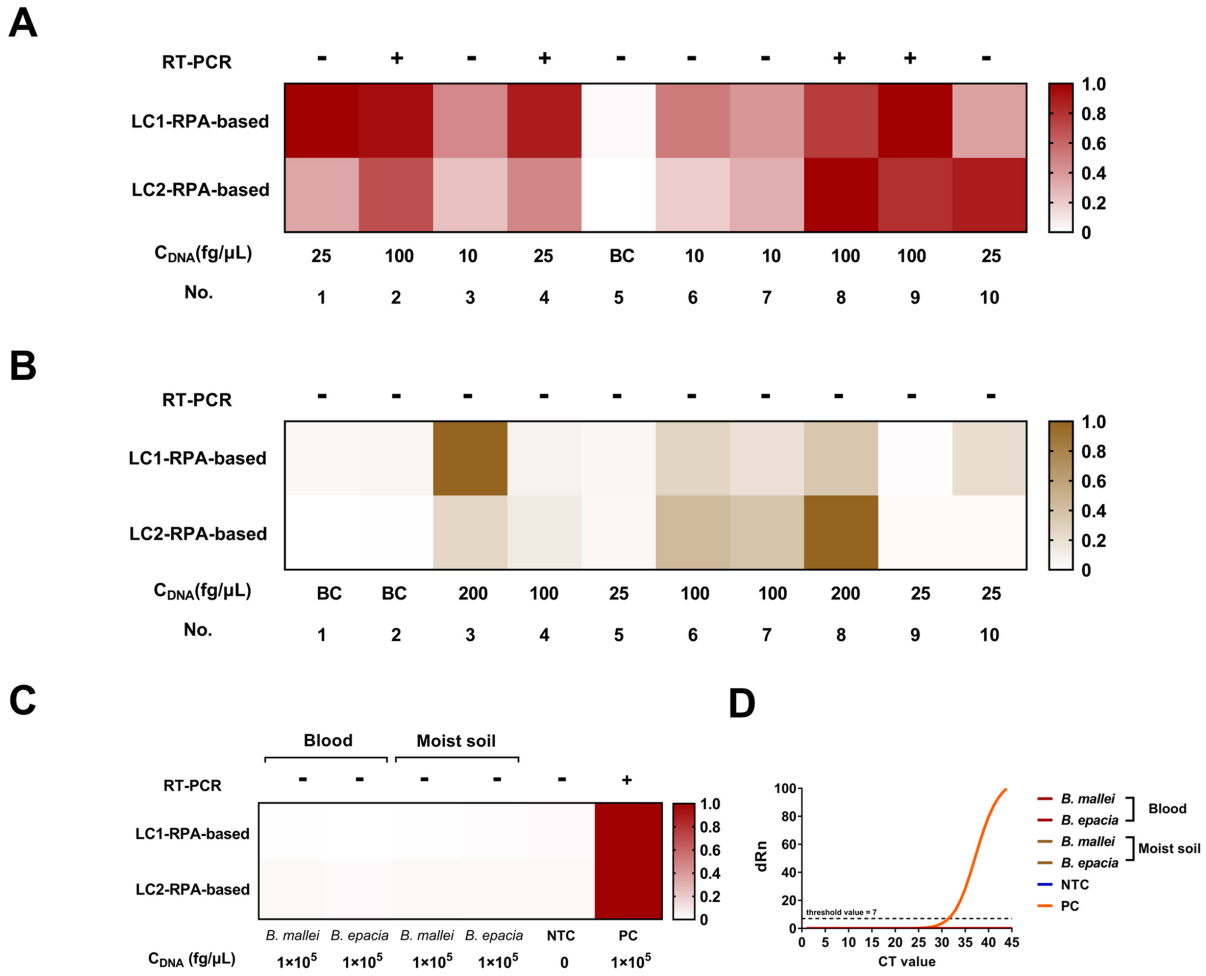
Blood and moist soil sample tests by the dual-target RPA-CRISPR/Cas12a assay and the RT-PCR assay, concentration of DNA, C_DNA_. **(A)** Blood samples test. **(B)** Moist soil samples test. **(C)** Analyze the potential cross-reacting bacterial DNA in the simulation experiment. **(D)** The RT-PCR assay.

The results of the dual-target RPA-CRISPR/Cas12a and RT-PCR assays for clinical and 12 environmental samples were shown in Figure 7. The LC1/LC2 RPA-CRISPR/Cas12a assay detected all positive blood samples. However, the RT-PCR assay, only detected No. 2, 4, 8, and 9 positive blood samples and could not effectively detect lower concentrations of the positive samples (Figure 7A). The test results showed that the LC1/LC2 RPA-CRISPR/Cas12a assay has significantly lower sensitivity for positive moist soil samples (Figure 7B, No. 5, 9, and 10), than for blood samples in detecting positive moist soil samples (Figure 7A, No. 1, 4, and 10). The RT-PCR assay failed to detect all positive environmental samples. This might be attributed to the low extraction efficiency of small quantities of target DNA spiked in complex samples. Furthermore, the RT-PCR assay and the dual-target RPA-CRISPR/Cas12a assay did not have cross-reactions with *B. mallei* and *B. cepacia* (Figures 7C,D) in two simulated samples. Therefore, whether in blood samples or more complex soil samples, the dual-target RPA-CRISPR/Cas12a assay showed superior detection results to the RT-PCR assay. More importantly, the dual-target RPA-CRISPR/Cas12a assay had the advantage of a short detection time, which facilitated on-site testing of melioidosis.

## 4. Discussion

*B. pseudomallei* is a gram-negative bacterium found in soil and water in tropical and subtropical regions of the world (33, 34). *B. pseudomallei* causes melioidosis, as a potential bioterror agent, that poses a threat to biosecurity. In addition to being a human pathogen, *B. pseudomallei* can infect and cause disease in cattle, pigs, goats, horses, dolphins, koalas, kangaroos, deer, cats, dogs, and gorillas. A report had called for action: time to recognize melioidosis as a neglected tropical disease (35).

A more rapid and sensitive detection method is required for monitoring *B. pseudomallei* infection and for the prevention and treatment of melioidosis. Various methods (8) have previously been attempted to identify *B. pseudomallei*. However, it is difficult to distinguish *B. pseudomallei* from *Burkholderia* and other closely related species due to their high phenotypic and genetic similarity. To remedy these deficiencies, here we had obtained 44 specific sequence tags of *B. pseudomallei* by bioinformatic analysis. Two of these tags on chromosome 1 and chromosome 2 of *B. pseudomallei* were used to develop a dual-target detection method. Notably, the selection of dual targets on core genomes (genes present in all strains) of two chromosomes were more conservative, stable, and less prone to horizontal gene transfer because *B. pseudomallei* is a high-frequency recombinant bacterium (36). The other 42 specific sequence tags will also provide important clues for the development of molecular diagnostic techniques for melioidosis in the future.

The RPA primers and crRNAs we designed were based on the selected sequences of *B. pseudomallei*, respectively. RPA specifically amplified the pathogen target, and then the crRNA/Cas12a/amplification product formed a ternary complex. Studies have shown that Cas12a has a single-base recognition ability, which gives the technology high specificity (31). Depending on the advantages of specificity, rapidity, ultrasensitivity, and covering two chromosomes, the dual-target RPA-CRISPR/Cas12a assay developed in this study will play an important role in the accurate and rapid diagnosis of *B. pseudomallei* in clinical and field, and will also fill the gap in CRISPR-based vitro diagnosis of melioidosis, which serves as a valuable reference for subsequent research.

## 5. Conclusion

We identified 44 specific sequence tags from the core genome sequences of chromosomes 1 and 2 of *B. pseudomallei* by bioinformatics methods, and two of them were used to develop a dual-target RPA-CRISPR/Cas12a detection method for highly specific identification of *B. pseudomallei*. The specific, rapid, and ultrasensitive detection, as well as the inclusion of both chromosomes, will allow the dual-target assay to play an important role in the accurate and rapid diagnosis of *B. pseudomallei* in clinical and field settings, as well as improving CRISPR-based *in vitro* diagnosis of melioidosis. Our study therefore has great potential for *B. pseudomallei* detection and the prevention and treatment of melioidosis. In summary, our study enriches the potential of *in vitro* diagnosis of pathogenic bacteria based on CRISPR. In addition, this study can be extended to other pathogenic bacteria detection applications, especially when phenotypic and genetic similarity makes it difficult to distinguish between the same genus and the same species of pathogenic bacteria. However, more work needs to be done to apply the technology to practical applications, such as nucleic acid-free extraction and the development of integrated microfluidic detection.

## Data availability statement

The data has been successfully deposited on NCBI and the Bioproject number is PRJNA930628. Additionally, the raw data for figures is provided in the Supplementary information.

## Author contributions

YY and X-L-LZ designed the study. J-XZ and YY wrote the manuscript. J-XZ, J-HX, and BY constructed CRISPR-Cas12a detecting platform. X-L-LZ and J-XZ performed bioinformation analysis. BY and X-DW performed DNA extraction and RT-PCR of samples. YY, J-XZ, and J-HX analyzed the data. X-hM provided some *B. pseudomallei* genomic DNA. YY and J-LW contributed project administration. All authors contributed to the article and approved the submitted version.

## Conflict of interest

The authors declare that the research was conducted in the absence of any commercial or financial relationships that could be construed as a potential conflict of interest.

## Publisher’s note

All claims expressed in this article are solely those of the authors and do not necessarily represent those of their affiliated organizations, or those of the publisher, the editors and the reviewers. Any product that may be evaluated in this article, or claim that may be made by its manufacturer, is not guaranteed or endorsed by the publisher.

## Supplementary material

The Supplementary material for this article can be found online at: https://www.frontiersin.org/articles/10.3389/fpubh.2023.1153352/full#supplementary-material

## Footnotes

^1^https://www.ncbi.nlm.nih.gov/bioproject/PRJNA930628

^2^https://www.ncbi.nlm.nih.gov/genome/browse#!/prokaryotes/476/

^3^https://www.ncbi.nlm.nih.gov/genome/browse#!/prokaryotes/477/

^4^http://hannonlab.cshl.edu/fastx_toolkit/

^5^https://github.com/tseemann/shovill

^6^http://mummer.sourceforge.net/

^7^https://cme.h-its.org/exelixis//web/software/raxml/

^8^https://github.com/tseemann/prokka

^9^https://sanger-pathogens.github.io/Roary/

^10^https://blast.ncbi.nlm.nih.gov/Blast.cgi
