# Supplementary material for: Detection of Burkholderia pseudomallei with CRISPR-Cas12a based on specific sequence tags
Source: Front Public Health. 2023 Apr 28;11:1153352. doi: 10.3389/fpubh.2023.1153352 (PMC10211466; doi:10.3389/fpubh.2023.1153352)
Supplement: Supplementary file 1 [file data_sheet_1.pdf]

Supplementary Figure S1. Evolutionary relationships among the genus *Burkholderia*, including 11 genomes of seven species. The rooted tree was constructed by using maximum likelihood.

Supplementary Figure S2. (A) The amplification curve of RT-PCR, Y-axis: dRn = Rn (sample) - Rn (Blank), Rn = R/RROX. (B) Standard curve developed using 10-fold serial dilution of DNA from B. pseudomallei ranging from 1 ng to 100 fg of genomic DNA. Error bars represent mean ± SEM, where n = 2 replicates.

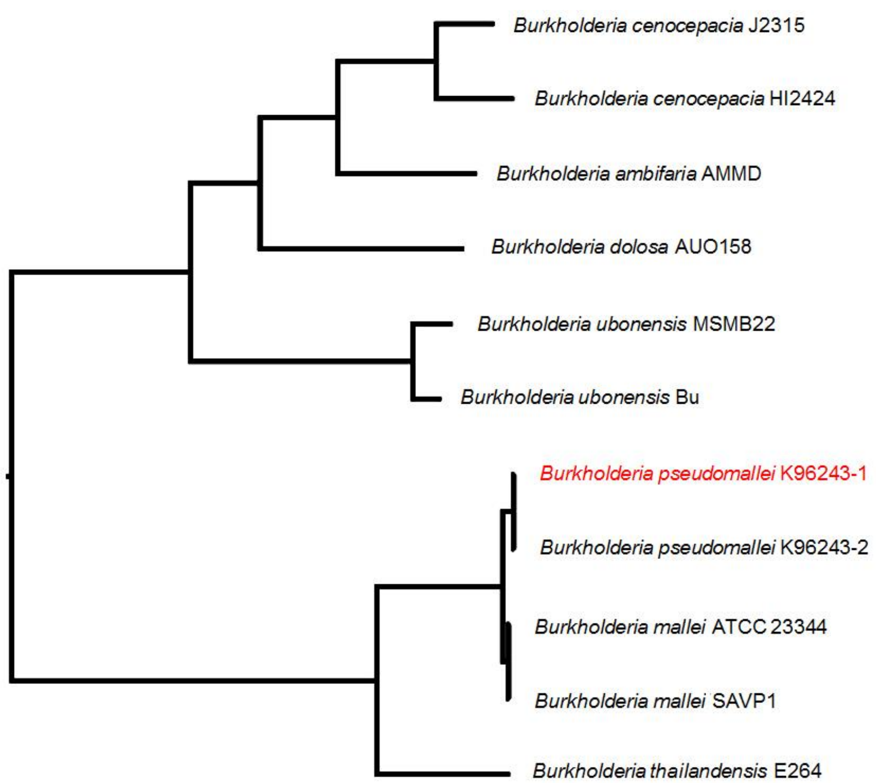

Supplementary Figure S1.

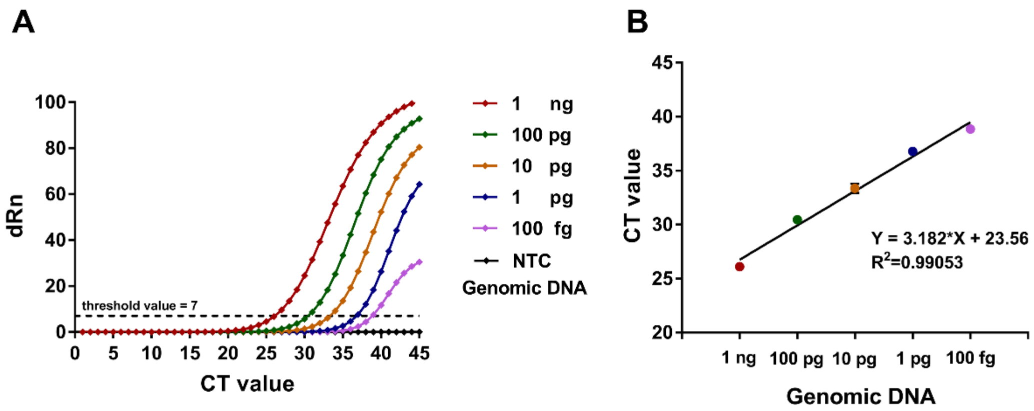

Supplementary Figure S2.
